# Assessing hospitals' clinical risk management: Development of a monitoring instrument

**DOI:** 10.1186/1472-6963-10-337

**Published:** 2010-12-13

**Authors:** Matthias Briner, Oliver Kessler, Yvonne Pfeiffer, Theo Wehner, Tanja Manser

**Affiliations:** 1 ETH Zurich, Center for Organizational and Occupational Sciences, Kreuzplatz 5, 8032 Zurich, Switzerland; 2 Lucerne University of Applied Sciences and Arts, Lucerne School of Business, Zentralstrasse 9, 6002 Lucerne, Switzerland; 3 University of Fribourg, Department of Psychology, Rue P.-A. de Faucigny 2, 1700 Fribourg, Switzerland

## Abstract

**Background:**

Clinical risk management (CRM) plays a crucial role in enabling hospitals to identify, contain, and manage risks related to patient safety. So far, no instruments are available to measure and monitor the level of implementation of CRM. Therefore, our objective was to develop an instrument for assessing CRM in hospitals.

**Methods:**

The instrument was developed based on a literature review, which identified key elements of CRM. These elements were then discussed with a panel of patient safety experts. A theoretical model was used to describe the level to which CRM elements have been implemented within the organization. Interviews with CRM practitioners and a pilot evaluation were conducted to revise the instrument. The first nationwide application of the instrument (138 participating Swiss hospitals) was complemented by in-depth interviews with 25 CRM practitioners in selected hospitals, for validation purposes.

**Results:**

The monitoring instrument consists of 28 main questions organized in three sections: 1) Implementation and organizational integration of CRM, 2) Strategic objectives and operational implementation of CRM at hospital level, and 3) Overview of CRM in different services. The instrument is available in four languages (English, German, French, and Italian). It allows hospitals to gather comprehensive and systematic data on their CRM practice and to identify areas for further improvement.

**Conclusions:**

We have developed an instrument for assessing development stages of CRM in hospitals that should be feasible for a continuous monitoring of developments in this important area of patient safety.

## Background

Managing the unexpected is an essential everyday concern in high-risk organizations such as hospitals [1]. Modern medicine has led to increasingly complex forms of treatment and processes of care. This results in a range of opportunities for improved care, but also increases the risk of adverse events and patient harm. Risks associated with patient care can never be completely eliminated, therefore, clinical risk management plays a crucial role in enabling hospitals to enhance patient safety [2].

Risk management (RM) generally encompasses risks in the political, legal and business environment [cf. [3,4]]. *Clinical risk management *(CRM) is a specific form of RM focusing on clinical processes directly and indirectly related to the patient. Therefore, we define CRM as all structures, processes, instruments and activities that enable hospital employees to identify, analyze, contain and manage risks while providing clinical treatment and patient care [cf. [5,6]]. Due to this focus, aspects of overall hospital governance (e.g. financial or infrastructural RM) or health policy issues (e.g. accreditation) were not considered in developing the monitoring instrument, although they do influence patient safety. Similar to the concept of ''safety management systems'' [cf. [7-9]], systematic CRM integrates both proactive and reactive approaches and frames the hospital as a system, instead of focusing on individuals and their potential for committing errors [10-12].

Although hospitals were always concerned with augmenting safety, it has only been since the Institute of Medicine reports "To err is human" [13] and "Crossing the quality chasm" [10] that a widespread application of systematic CRM has been considered [2,11,14]. At the organizational level, many RM tools have been adapted from other high-risk industries such as aviation. A prominent example is incident reporting, which is gaining increased acceptance among hospitals and is viewed as a possible method to promote learning from incidents [15-17]. At the national and international level, several patient safety initiatives have been launched [18-20].

Despite the multitude of programs, initiatives, and tools that can all be seen as elements of CRM, there is a lack of knowledge concerning their implementation in hospitals. Evidence exists on the implementation of outcome measurement related to patient safety (morbidity rates, complications, medication errors etc.). But to develop and implement CRM interventions successfully and to monitor their progress over time, hospitals require systematic data on their strengths and weaknesses [21]. Our primary aim was to develop an instrument that allows for continuous monitoring of the current state and planned developments of CRM in hospitals. As hospitals are rather decentralized and fragmented with regard to organizational strategies, structures, and culture [cf. [13]], the instrument differentiates between various services within a hospital. Therefore, the instrument provides a more accurate view of CRM in both the hospital as a whole and for services within the hospital.

At the national level, policy makers could use the results of such systematic monitoring to establish transparency, support change, and coordinate different CRM related programs. This is especially important with regard to the Swiss healthcare context. Swiss hospitals are governed rather autonomously by the 26 cantons (political units) and little national regulation takes place [22]. Therefore, many tools and initiatives are developed independently and implemented locally. Within the Swiss system, limited resources are spent on quality assurance (two to three-tenths of a percent), accreditation and certification is mostly voluntary and an integrative solution is not yet in sight. SanaCERT, the former Swiss Society of Quality in Healthcare, has certified 15 Swiss hospitals since 2003. Other hospitals were certified through International Organization for Standardization, European Foundation for Quality Management or the Joint Commission on Accreditation of Healthcare Organizations [23]. In this context, nationwide CRM monitoring could provide valuable data needed to effectively support local, regional and national improvement efforts.

## Methods

**Figure 1:**
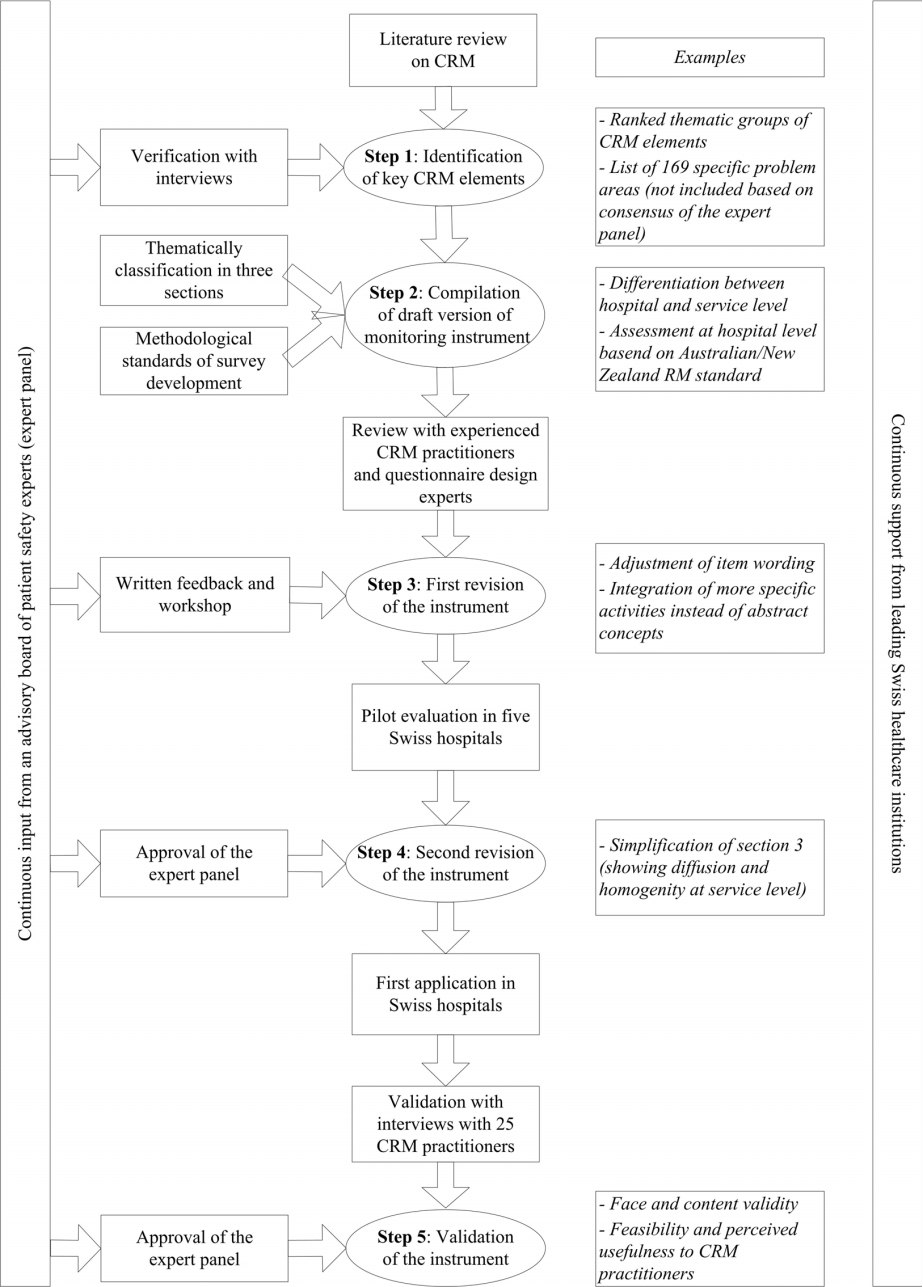
Procedure used to develop the monitoring instrument

The development of the monitoring instrument followed five steps with support from five Swiss healthcare institutions (specified in the acknowledgment section) and continuous input from an advisory board of eleven patient safety experts. This expert panel included the persons in charge of patient safety and/or quality of the five Swiss healthcare institutions, the president of the Swiss Society for Quality Management in Health Care, the head of quality of a major reinsurance company, and four clinical experts with a proven record of accomplishment in patient safety. Figure 1 shows an overview of the development process. Examples provided below illustrate the contribution of each step to the development of the monitoring instrument.

### Step 1: Identification of key CRM elements

The initial monitoring instrument was based on an inventory of important CRM elements derived from a literature review. Bibliographic databases (Medline, Psychinfo, ISI Web of knowledge), library catalogues and the internet were searched for citations of "CRM", "RM", "patient safety", "quality management" and "incident reporting" in titles, abstracts and key-words of scientific papers written in English or German. The publication period was not limited (termination of search: June 2007). The CRM elements found in the literature were sorted into thematic groups and ranked in order of relevance (i.e. times mentioned in literature). These CRM elements were verified using semi-structured interviews with the members of our expert panel focusing on completeness and relevance of the elements. The key CRM elements identified through this process were a systematic approach to clinical risks and patient safety, the implementation of the RM process, leadership, staff participation, safety culture, learning from incidents or errors, and education and training. Table 1 shows these elements and the corresponding questions in the instrument. Based on advice from our expert panel we did not include specific problem areas (so called "hot-spots" [24]) including medication errors, falls, pinprick injuries, etc., as the CRM processes and structures are generally the same for all these issues.

**Table 1:** Most important elements of CRM

Element	Rating	References	Questions in the instrument
*Systematic approach to clinical risks and patient safety*	Key requirement for CRM	[2,39,40,55]	Q1, Q3, Q9-Q13, Q24
*Implementation of the RM process*	Key requirement for systematic CRM	[6,40,56]	Q15, Q23
*Leadership*	Necessary condition for successful execution of CRM	[2,5,13,57-59]	Q16, Q26
*Participation of staff*	Necessary condition for successful execution of CRM	[13,40,56,58]	Q14, Q16, Q26
*Safety culture*	Necessary condition for blame-free CRM	[5,13,21,58,60,61]	Q14, Q26
*Learning from incidents or errors*	Compulsory as not to repeat mistakes	[2,15,16,62]	Q19, Q20, Q21, Q28
*Education and training*	Knowledge and skills need to be regularly updated	[13,21,40,63]	Q6, Q14, Q27

### Step 2: Compilation of draft version of the monitoring instrument

In the second step, we compiled a draft of the monitoring instrument. The key elements of CRM identified in step 1 were thematically classified into three sections (table 2) and survey questions were developed according to methodological standards in the social sciences [cf. [25,26]].

#### Section 1) Implementation and organizational integration of CRM

The first section examines how and to what extent CRM is embedded in existing organizational structures. It contains questions on organizational integration, resource allocation and professional background in relation to CRM in the hospital. Additional questions focus on environmental factors and constraints concerning CRM (e.g. regulatory frameworks).

#### Section 2) Strategic objectives and operational implementation of CRM at hospital level

The second and main section addresses the strategic and operational objectives of the hospital, the potential for optimization with regard to key CRM elements and the current state of CRM (implementation of the RM process and questions on leadership, staff participation and training) within the hospital. This section also contains a "focal theme" (i.e. the implementation of incident reporting systems because they are currently one of the most widely used CRM tools [2]) that may be changed in future applications of the instrument depending on developments in the field and trends in CRM.

To assess the implementation of the RM process at hospital level (see additional file 1, Question 15 (Q15)) we used the Australian/New Zealand RM standard [27]. We considered it as one of the most comprehensive standards for RM and most applicable to the hospital environment. This standard describes management of risks as an integral part of good governance, and states that RM is best embedded into existing organizational practices or business processes without favoring a specific management approach. It also emphasizes communication within and across all organizational units. It offers a systematic, proactive and integrative approach that can be adjusted to size of the organization, implementation possibilities and financial restrictions [27,28]. The Australian/New Zealand RM standard has also been integrated in the recent ISO/DIS 31000 RM norm [29]. The main elements are as follows [cf. [27,29]]:

1) *Risk strategy: *establish the external and internal RM context, develop criteria, and define the structure of RM.

2) *Risk identification: *Identify what, when, where, how, and why events can happen.

3) *Risk analysis: *Determine consequences and likelihood of possible events and the level of risk.

4) *Risk evaluation: *Compare risks against criteria and set priorities. Decide on the extent and nature of required actions.

5) *Risk treatment: *Identify and assess options. Prepare and implement action plans.

These five phases form an iterative process of continuous improvement that is supported by communication and documentation and is integrated into the risk monitoring process.

#### Section 3) Overview of CRM in different services

The third section focuses on key CRM elements in relation to their implementation in various hospital services (CRM process, communication and information, documentation, learning and developing, training, local incident reporting systems). As mentioned, this differentiation between hospital level (see section 2) and service or department level allows for a more precise view on the current situation and the planned developments of CRM within the fragmented organizational structure of hospitals.

### Step 3: First revision of the instrument

To improve the draft version of the instrument and to adjust it to the needs of CRM practitioners (persons responsible for CRM), we discussed the content, completeness and comprehensibility of the whole instrument with six experienced CRM practitioners in 60-90 minute interviews. The interviewees (five persons responsible for CRM in major Swiss hospitals and the CEO of a risk management company and president of the Swiss risk-management network) were selected according to suggestions from our advisory board. In addition, questionnaire design experts reviewed the draft version regarding wording, structure and order of questions and response alternatives. This was followed by written feedback and a workshop with our expert panel. Their input resulted in further precision in wording and more questions on specific activities with examples instead of abstract concepts or attitudes. An intense discussion took place of how to gain the overview of CRM in different services (section 3 of the instrument). One option was to include a separate questionnaire for every service; however, this would have been very difficult logistically, especially for larger hospitals. The final consensus was to ask the central CRM practitioner about the level of implementation of CRM elements within each service. The draft version of the monitoring instrument was adjusted according to the input from CRM practitioners and prepared for a pilot evaluation.

### Step 4: Second revision of the instrument

The pilot evaluation of the instrument was conducted with CRM practitioners from five Swiss hospitals differing in size and language (one university hospital, one regional hospital, one hospital group, one rural hospital, one private hospital), thus testing the applicability of the instrument in different organizational contexts. The CRM practitioners completed the instrument while thinking aloud. Two observers noted the remarks and discussed them afterwards with the respondents. This led to further minor adjustments of the monitoring instrument. Whereas all pilot evaluation participants perceived the content (i.e. key CRM elements) to be clear and complete, the format for giving an overview of CRM in different services (section 3) turned out to be difficult, as CRM practitioners often lacked the detailed knowledge about the level of implementation of these elements within each service. Therefore, the format was simplified, showing diffusion and homogeneity by asking whether key CRM elements were implemented in or planned for all services, certain services only, or none of the services. The expert panel then approved the final instrument.

### Step 5: Validation of the instrument

The monitoring instrument was first applied in Swiss hospitals in the winter of 2007/2008. This survey included open text fields (see additional file: A1, A2) where the participants could suggest improvements to the instrument, mention additional themes and ask questions. From 324 contacted hospitals, 138 completed the survey (43%). To validate the instrument, we conducted in-depth interviews with a purposeful sample of 25 CRM practitioners from May to September 2008. Information-rich cases were selected on the basis of the following criteria: developmental stages of CRM as assessed with the instrument, hospital type, hospital size, and language region [cf. [30]]. Interviews focused on a structured review of the survey results in comparison to their subjective perspective on CRM in their hospital and future developments pertaining to CRM. The interviews were audio-recorded for review and discussion in the research team. Feedback from the open text fields and the interviews was mostly positive for the instrument's content and design. The instrument was viewed as understandable, adequately complex and meeting the needs of CRM practitioners. The 25 interviewees found the instrument helpful to evaluate CRM strengths and weaknesses of their own hospital. Furthermore, participants emphasized that the instrument had a sensitizing effect for their daily practice (e.g. highlighting new areas of CRM, encourage discussion within management and staff). There were also some suggestions to modify the monitoring instrument (e.g. clearer definition of technical terms in form of a glossary, possibly a specialized section for different hospital types, and changing focal themes over time), but no requirements for fundamental modifications. Therefore, the expert panel approved the monitoring instrument for a second application in Switzerland in 2010.

## Results

### Content of the monitoring instrument for CRM

The final monitoring instrument developed in this study consists of 28 main questions (in parts with sub-questions) and takes approximately 60 minutes to complete. It allows for the assessment of the current state and planned developments of CRM in hospitals. It should be completed by the clinical risk manager/person responsible for CRM of the hospital but can also be completed with input from her/his CRM team. Questions are designed to be applicable for hospitals of various types (i.e. university hospital, acute-somatic hospital, psychiatric hospital, rehabilitation centers) and different size. They can be used for repeated assessments to track changes over time and thus allow for continuous monitoring. The monitoring instrument is available in a paper-pencil and in a web-based version and has been translated from its original language (German) into French and Italian for use in the three language regions in Switzerland. An English cross translation is also available and is presented in additional file 1.

Table 2 provides an overview of the three sections of the monitoring instrument as described in the methods section including the different themes, examples for each item and references to the questions in the instrument for the final version.

**Table 2:** Thematic overview of the content of the monitoring instrument

Section	Themes	**Examples for item content**:	Instrument
*Section 1: Implementation and organizational integration of CRM*	Organizational integration	Is there a designated person responsible for the central coordination of CRM activities in your hospital?	Q1
		In which organizational unit is he integrated (member of the hospital board, staff position on the hospital board, integrated into the individual services, ...)?	Q2
		Is there... ... a written job description, ... a separate budget for CRM, ...	Q3
	Resource allocation	Responsibilities; staff size	Q4-Q5
	Professional background	Medicine, nursing professionals, business administration, etc	Q6
	Environmental factors and constraints	Political or legal frameworks	Q7-Q8
*Section 2: Strategic objectives and operational implementation of CRM*	Strategic and operational objectives	Strategic objectives of the hospital, and especially of CRM	Q9-Q11
		Annual operational objectives of CRM	Q12-Q13
	Optimization potentials with regard to key CRM elements	We need... ...more continuing training in CRM, ...more standardized procedures, and so on	Q14
	Current state of CRM	Implementation of the CRM process	Q15
		Questions about leadership, participation of staff and training	Q16
		Strengths and needs of CRM	Q17-Q18
	Focal theme: Incident reporting system	Distribution, implementation and character of the system	Q19-21
*Section 3: Overview of CRM in different services *(Q22: number of services)	CRM Process	Service-internal tasks, competences and responsibilities in CRM are clearly defined.	Q23
	Communication and information	There are guidelines to ensure that patients are informed before treatment about possible risks.	Q24
	Documentation	Medical records are managed electronically.	Q25
	Learning and developing	The service's board of directors takes clinical risks into account when organizational changes are implemented.	Q26
	Continuing education/training/advanced training	Staff receive advanced training in effective teamwork strategies.	Q27
	Focal theme: Local Incident Reporting Systems	Standardized procedures are applied for the cause analysis of reported incidents.	Q28

### Rating scales of the monitoring instrument for CRM

Different rating scales were used in the monitoring instrument depending on the type of question. To assess the hospitals' current situation as well as plans for developments related to CRM a three-point rating scale ("yes", "planned", "no") indicating the presence, absence or planned development of a particular element (e.g. written strategic objectives) was used (see Q1, Q3, Q9, Q10, Q12).

To capture the development stages at hospital level (Q15, Q16, Q20) several possibilities were considered such as the five levels of safety culture advancement reaching from pathological to generative [31] or the design safety capability maturity model that also contains five levels reaching from uncontrolled to optimized [32]. We decided to use the transtheoretical model (TTM) because it is process-oriented and therefore sensitive to change and well-suited for a monitoring instrument. The TTM was originally developed to allow a description of behavioral change at the individual level [33] and has since been applied to describe organizational change [34-36]. According to the TTM, organizations pass through five different stages until a change becomes permanent: Precontemplation, Contemplation, Preparation, Action and Maintenance (see table 3). These stages represent a continuum of readiness to take and sustain action and allow for a detailed investigation on how organizations change [37]. The description of the stages had to be adapted as changes are more complex in organizations (compared to individual changes) and usually need more time. In the "precontemplation stage", a CRM element is not yet examined and therefore no action is intended. In the "contemplation stage", the element is already examined but no plans are made yet, whereas in the "preparation stage" the implementation of the element is planned in the next 12 months. In the "action stage", the element is implemented although not systematically, which is often the case in hospitals as an element is implemented only partially. Only in the most mature "maintenance stage" systematic implementation is reached. To cover those hospitals that intentionally decided against implementing the proposed element we added another category labeled "Deliberate decision against implementation" in the maintenance stage. The importance of such a category was shown in the Precaution Adoption Process Model where it is labeled "Decided not to act" [38].

**Table 3:** Stages of change in the transtheoretical model [according to 37]

Stage	Behavioral change	Adapted to measure the development stages at hospital level
Precontemplation (Stage 1)	Not intending to take action within the next 6 months	Not yet examined
Contemplation (Stage 2)	Intending to take action within the next 6 months	Examined, but so far no implementation plan
Preparation (Stage 3)	Intending to take action in the next 30 days	Implementation planned in the next 12 months
Action (Stage 4)	Made overt changes less than 6 months ago	Not systematically implemented
Maintenance (Stage 5)	Made overt changes more than 6 months ago	Systematically implemented/ Deliberate decision against implementation

To capture variations in the implementation of key CRM elements at service level we used a rating scale showing diffusion and homogeneity: "true for all services", "true for certain services", "planned for all services", "planned for some services" and "not true for any service" (Q23-Q28). Multiple answers were possible (e.g. to account for elements that are already implemented in certain services and planned for all services). To measure the level of agreement concerning optimization potential a four-point Likert scale was used ("not at all true", "not quite true", "quite true", and "true"; Q14). Participants were also asked to fill in open text fields to provide specific information on their CRM practice, for example on the strengths of a particular CRM system (e.g. Q7, Q8, Q17, Q18, Q21).

## Discussion

Patient safety and CRM have become topics of great importance in recent years but are still evolving slowly [39]. So far, many hospitals have performed a range of activities aiming at enhanced patient safety but CRM is seldom approached systematically [cf. [40]]. The monitoring instrument developed in this study attempts to fill that gap by providing a systematic and comprehensive overview of CRM and a baseline for future developments by monitoring the current situation and planned developments of CRM in hospitals [21].

By identifying key CRM elements in the literature, compiling the questions and reviewing the monitoring instrument with experts, the study also contributed to the definition of what constitutes CRM both conceptually and in practice. Since there is not yet agreement on how to best implement CRM, the instrument accounts for different managerial approaches (e.g. centralized vs. decentralized) with selected questions in the instrument (Q1-5) and makes an explicit distinction between hospital and service level. Although compiled from healthcare-related literature, the content of the instrument is in line with the most critical elements applied in safety management systems as described by other high risk industries, such as oil and gas [41] or aviation [42]. These include a sound safety policy (Q10-Q13 in the monitoring instrument), a clear distribution of responsibilities at all management levels (Q1-Q3), adequate allocation of resources (Q4-Q6, Q14), leadership and involvement of people (Q16, Q26), learning from experience through incident investigation (Q20, Q28), effective safety training (Q27) and assessing and controlling of risks (Q15, Q23).

To assess the development stages at hospital level, a rating scale based on the TTM was used, as it precisely describes an organization's development stages and thus, provides useful information for hospital self-assessment and for comparisons between hospitals. Furthermore, the TTM supports the design and implementation of interventions targeting particular organizational development stages and therefore, is useful to "reduce resistance, increase participation, reduce dropout and increase change progress among employees" [[36], p. 247]. This is essential to any organizational change strategy as top-down implemented change strategies are often destined to fail, because they may not consider employees' and thus organizations' stage-dependent willingness to change. Stage models for measuring development have also been applied in other areas of patient safety research, for example in evaluating the development and maturation of organizational safety culture [31]. Current examples are the quality improvement maturity index [43] or a framework for exploring organizational readiness for success in organization-wide patient safety improvement programs [44].

### Benefits of the monitoring instrument

The monitoring instrument provides several benefits at the hospital, service and national level. It allows hospitals to achieve comprehensive and systematic data on their CRM by delivering an individual assessment of each participating hospital. For benchmarking purposes, all participating hospitals received a feedback document showing the aggregated answers of all surveyed hospitals and the aggregated results for their own hospital type (i.e. university hospital, acute-somatic hospital, psychiatric hospital, rehabilitation centers). The answers of the hospital itself were highlighted to identify the hospital's position in the field and to obtain a CRM profile showing the hospital's strengths and weaknesses. This profile may aid in prioritization, development and implementation of interventions tailored to the development stages of a particular hospital. The possibility to assess and continuously monitor CRM elements and exchange and compare these data across hospitals of similar type can promote learning and sharing of good CRM practices. Additionally, the feedback document allows support on a CRM action plan because of its systematic approach. Thus, the monitoring instrument can serve as an inventory that may guide CRM development.

The overview of CRM at service level demonstrates diffusion and homogeneity of the implementation of CRM elements. This offers a basis for exchange between centralized and decentralized CRM units and the possibility to systematize CRM elements hospital-wide as central strategies. Similarly, local initiatives can be combined and adjusted, especially in large hospitals.

The usefulness at national level depends on the political context. The main aims for CRM in Switzerland are gaining transparency (e.g. in the identification of well-established and problematic CRM practices), supporting exchange, and coordinating different CRM interventions. However, a central regulation system could encounter much resistance due to the political structures.

In addition, an increased awareness for CRM was observed during the survey even in non-participating hospitals. Many hospitals stated that they were not ready yet, but wanted to participate in the future, and some hospitals offered exploratory interviews.

### Limitations

The instrument described here focuses on the evaluations of the hospitals' clinical risk manager, whose assessment may be different from perceptions of individual clinicians in the hospital. Furthermore, self-assessment can generate quite different responses, if the risk manager thinks that drawing attention to gaps may result in allocation of increased resources or if gaps may be viewed as a failure on his or her part. The monitoring instrument offers two ways to deal with this potential bias. First, the questions refer to the responsibility of the hospitals' clinical risk manager and focus on factual issues instead of attitudes or motives. Second, hospitals were encouraged to discuss questions internally. About 50% of the interviewed CRM practitioners indicated that a discussion took place between them, the CEO and other relevant personnel and that they agreed on the most adequate answer for their institution.

The benefit of measuring development stages may be debatable. For example, it can be questioned if an unsystematic implementation (stage 4) of a CRM element is more mature than if the hospital-wide implementation of this CRM element is planned in the next 12 months (stage 3). One can even debate if there is something like a "development stage" or a "maturity index" [45] of CRM at hospital level, as some hospitals might predominantly use local CRM tools or approaches. The monitoring instrument accounts for those local specifications with an overview of CRM in different services in section 3. The question remains whether it is possible to assess CRM at service level via the central risk manager. Results of the empirical application will allow a deeper understanding of the development stages of CRM and their progression in hospitals.

As the literature review was conducted to identify the most important CRM elements and not all possible CRM elements, it was goal-oriented and not conducted following guidelines for systematic reviews [cf. [46,47]]. Since the completion of our review in June 2007 more literature on CRM has been published and a systematic review might identify some additional elements. However, for this study, the verification of relevance and completeness of the included elements with members of the expert panel affirms a solid basis for the development of the instrument. As the monitoring instrument is not an empirical or clinical test, communicative validation, practicability and acceptance in the field are all very important. The review of the monitoring instrument with experts assured that it possesses both content and face validity. The interviewed CRM practitioners viewed the instrument mostly as being practical and matching their needs. The response rate of 43% of all Swiss hospitals in the first application demonstrates that the acceptance of the monitoring instrument was good, as participation was voluntary. Another sign of acceptance was that two thirds of the respondents were interested in being interviewed subsequent to the survey and some hospitals even contacted us to become future research partners.

Finally, some suggestions to optimize the monitoring instrument have not yet been implemented. Regarding definitions of terms in a glossary, the Swiss Hospital Association H+ (a partner of our project) provides a glossary of quality terms in German on their website [48]. A specialized section for different hospital types was discussed controversially as it may be helpful at the local level but make generalizations and strategic planning at the regional or national level more difficult.

## Conclusions

The second application of the monitoring instrument took place in 2010 and the results of both studies will be reported in later publications. Simultaneously the Institute for Patient Safety of Germany has conducted a nationwide survey on CRM for the first time using large parts of this instrument [49]. This first use of the instrument in international context opens up the possibility to compare CRM in healthcare systems of different countries. Also, using the monitoring instrument in regular intervals will allow to identify and track changes, developments and emerging trends of CRM over time for hospitals in general, for specific hospital types and for each hospital individually. This is important as CRM will move forward based on the availability of new instruments, knowledge, technologies, innovations and developments [cf. [10,40,50]]. We suggest at least one year between applications, as changes in hospitals need time, and the rating scales in our instrument ask about periods of one year.

In Switzerland, hospital accreditation of RM has been voluntary up to this point. As this might change in the future, this instrument could be used as part of a comprehensive RM assessment [51]. It could also be integrated into existing certification systems, for example in the established SanaCERT certification of quality standards [52].

Finally, monitoring CRM focuses primarily on resources, structures, and processes, but CRM eventually aims at enhancing patient safety and improving medical services and care. To investigate the link between CRM and patient safety, any monitoring instrument should be correlated with data on clinical outcomes and with other empirical data (e.g. quality management). This is a very complex venture, as one has to account for the different sources and methods to gather data, hospital-specific case mixes, nature of critical incidents and so forth. For example, research in England suggested for six out of nine indicators that care might be getting less safe despite numerous initiatives to improve patient safety [53]. However, this result could be due to "improved coding" (p. 1206) and the authors believe that "the lack of reliable information on safety and quality of care is hindering improvement in safety across the world" (p. 1205). Similarly, research on the effectiveness of accreditation is "at an embryonic stage" and "no positive or consistent relationship between accreditation and clinical performance have been found" [[54], "The present status of research into accreditation", para. 1]. But as Vincent [2] states, CRM aims at reducing or eliminating harm to patients. Therefore, it is essential to systematically develop and implement tools such as this instrument, for evaluation.

## Abbreviations

CRM: Clinical risk management; RM: Risk management; Qxx: Question xx; TTM: Transtheoretical model;

## Competing interests

The authors declare that they have no competing interests.

## Authors' contributions

MB, OK, TM and YP were involved in designing the draft version of the monitoring instrument. All authors participated in the further steps to develop its final form. MB drafted the initial article and led the revising of the manuscript. TM, OK, YP and TW revised the manuscripts critically for important intellectual content. All authors read and approved the final manuscript.

## Pre-publication history

The pre-publication history for this paper can be accessed here:

http://www.biomedcentral.com/1472-6963/10/337/prepub

## Supplementary Material

Additional file 1 **English translation of the monitoring instrument for assessing hospitals' clinical risk management**. The additional file 1 shows the final monitoring instrument for CRM as developed in this study. It was cross-translated and discrepancies were eliminated. For an application in English-speaking countries it may have to be adjusted to the different healthcare contexts. Click here for file
