# Supplementary material for: Assessing hospitals' clinical risk management: Development of a monitoring instrument
Source: BMC Health Serv Res. 2010 Dec 13;10:337. doi: 10.1186/1472-6963-10-337 (PMC3022874; doi:10.1186/1472-6963-10-337)
Supplement: Additional file 1 — English translation of the monitoring instrument for assessing hospitals' clinical risk management. The additional file 1 shows the final monitoring instrument for CRM as developed in this study. It was cross-translated and discrepancies were eliminated. For an application in English-speaking countries it may have to be adjusted to the different healthcare contexts. [file 1472-6963-10-337-S1.DOC]

**Additional file 1 English translation of the monitoring instrument for assessing hospitals’ clinical risk management**

The additional file 1 shows the final monitoring instrument for clinical risk management (CRM) as developed in this study. It was cross-translated and discrepancies were eliminated. For an application in English-speaking countries it may have to be adjusted to the different healthcare contexts.

**Section 1)** **Implementation and organizational integration of CRM in your hospital**

**1A Organizational integration**

Q1 Is there a designated person responsible for the central coordination of CRM activities in your hospital?

 Yes, since ____________ (please specify year)

 Is planned in _______ months.

 No, is not envisaged.

The job title of this person in our hospital is: __________________________________

Q2 How are you (or, if applicable, the central CRM team) integrated into the hospital on an organizational level in regard to your/their CRM functions/activities? (tick one or more)

 member of the hospital board/administrative board.

 reporting directly to the hospital board/ administrative board (line function).

 a staff position on the hospital board/ administrative board.

 complete decentralized integration into the individual services/ departments.

 reporting to the following service (medical director, nursing director, central services, etc.):

____________________________________________________________________________________

 other integration within the organization? Please describe (responsibilities, service, level, etc.):

____________________________________________________________________________________

| Q3 For the functions/activities of the central clinical risk management (or for your own function) in your hospital is there/are there… | Yes | Planned over the next 12 months | No |
| --- | --- | --- | --- |
| … written job/task descriptions? |  |  |  |
| … detailed statements of work? |  |  |  |
| … the opportunity to initiate projects on your own? |  |  |  |
| … a delegation of authority allowing you to implement measures on your own? |  |  |  |
| … a separate budget to initiate activities and projects in CRM?  If so, what is the budget for this year? _____________________ |  |  |  |
| … regularly scheduled exchanges between individual services/departments? |  |  |  |
| … the opportunity to bring CRM issues to the attention of the hospital board?  In what form (please specify)? ___________________________________ |  |  |  |

**1B Resource allocation**

| Q4 What percent of your personal full-time equivalent (FTE) do you invest in the following activities? (actual time spent, 10% = ½ day) | Percentage of FTE, approx.: |
| --- | --- |
| Your total FTE percent | ______ % |
| *Percent of FTE in the following activities…* |  |
| … in *clinical* risk management | ____ % |
| … in *non-clinical* risk management (e.g. financial or technical risks) | ____ % |
| … in quality management of non-clinical risks (e.g. procedure optimization to increase patient satisfaction) | ____ % |
| … in all other activities not mentioned above (e.g. clinical and management activities, organization and staff development) | ____ % |

| Q5 What are the human resources currently available for CRM in your hospital (i.e. people with permanent CRM positions)? | Number of persons: | Percentage of FTE, approx.: |
| --- | --- | --- |
| How many people are currently working in the central CRM team of your hospital and what is the percentage of their FTE? | ________ | ________ % |
| How many people (and percentage of FTE) with permanent a permanent CRM-assignment are there in total in the *decentralized* CRM functions/teams (e.g. incident reporting delegate in the clinics/services/care centers)? | ________ | ________ % |
| Is an increase in CRM staff planned over the next 12 months?   No   Yes, *centralized* to the following extent:   Yes, *decentralized* to the following extent: | ________  ________ | ________ %  ________ % |

**1C Professional background**

| Q6 What elements of your professional background and of your basic and advanced training do you personally and, if applicable, do the members of the CRM team bring into your/their work? What competences are you planning to reinforce or implement in your central CRM team over the next 12 months? (tick one or more): | Applies to me | Applies to some others in the CRM team | Definitely planned over the next 12 months |
| --- | --- | --- | --- |
| Medical degree |  |  |  |
| Medical specialist FMH |  |  |  |
| Qualified nurse |  |  |  |
| Qualified medical technician |  |  |  |
| Qualified physical/occupational therapist |  |  |  |
| Degree in nursing sciences |  |  |  |
| Basic and/or advanced training in business administration |  |  |  |
| Basic and/or advanced training in psychology |  |  |  |
| Basic and/or advanced legal training |  |  |  |
| Advanced training in clinical risk management |  |  |  |
| Advanced training in quality management |  |  |  |
| Other (please specify): ___________________________ |  |  |  |

**1D Environmental factors and constraints concerning CRM**

Q7 In your opinion, which are the most important suggestions concerning the political or legal frameworks influencing CRM in hospitals?

(open text field) _____________________________________________________________________

Q8 Do you have any other comments on the implementation and organization of CRM in your hospital? Are there any special features not mentioned in the questions above?

(open text field) _____________________________________________________________________

**Section 2) Strategic objectives and operational implementation of CRM in your hospital**

**2A Strategic and operational objectives of your hospital**

Q9 Does your hospital have a formal written strategy?

 Yes

 In the planning stages, should be available in __________ months.

 No

Q10 Does your hospital have *formal written strategic objectives for CRM*?

 Yes 🡪 Where are these delineated? ____________________________________________________

 In the planning stages, should be available in __________ months.

 No

Q11 What are (or would be in your opinion) the 3 most important strategic CRM objectives of your hospital?

Strategic objective 1 (please specify; 3 open text fields): _________________________________

Q12 Have *annual operational CRM objectives* been established in your hospital?

 Yes 🡪 Where are these delineated? ____________________________________________________

 In the planning stages, should be available in __________ months.

 No

Q13 What are (or would be in your opinion) the 3 most important operational CRM objectives that can be met over the next 12 months?

Annual objective 1 (please specify; 3 open text fields): _________________________________

**2B Optimization potentials with regard to CRM key elements**

| Q14 To what degree are the following statements true in regard to your hospital? (please tick only one box per statement).  *In order to establish more effective CRM in our hospital, we need …* | Not at all true | Not quite true | Quite true | True | |
| --- | --- | --- | --- | --- | --- |
| … more clearly designated contact persons in the services / departments, who assure the liaison with central CRM (or with you). |  |  |  |  | |
| … more regular exchanges between central CRM (or you) and the individual services / departments. |  |  |  |  | |
| … more horizontal networking between the services / departments. |  |  |  |  | |
| … clearer regulations of tasks, competences and responsibilities (organizational and leadership structures). |  |  |  |  | |
| … more standardized procedures and processes (e.g. treatment guidelines, checklists, etc.). |  |  |  |  | |
| … a more open and honest way of dealing with errors and weaknesses within the system. |  |  |  |  | |
| … additional financial resources. |  |  |  |  | |
| … additional human resources.  Most importantly for the following functions (please specify):  Function 1: / Function 2: (open text fields) |  |  |  |  | |
| … more continuing training in CRM and patient safety.  Most importantly for the following staff groups: (please specify):  Staff group 1: / Staff group 2: / Staff group 3: (open text fields) |  |  |  |  | |
| … specific objectives for developing safety culture.  Objective 1: / Objective 2 (please specify): (open text fields) |  |  |  |  | |
|  |  |  |  |  | |
| Other optimization measures (please specify): _______________________________________________________ | | | | |  |

**2C Current state of CRM in your hospital**

| Please specify how systematically the following aspects of CRM are currently being implemented in your hospital*. (Note: in the questions below we are interested in general CRM; specific questions about critical incident reporting systems will follow further down.)* | Not yet examined | Examined, but so far no implementation plan | Implementation planned over the next 12 months | Not systematically implemented | Systematically implemented | Intentionally not implemented |
| --- | --- | --- | --- | --- | --- | --- |
| **Q15 Items on the implementation of the CRM-Process:** |  |  |  |  |  |  |
| Tasks, competences and responsibilities in the CRM of your hospital are defined. |  |  |  |  |  |  |
| Clinical RM procedures are defined and documented throughout the hospital. |  |  |  |  |  |  |
| Clinical risks are identified at hospital-level. |  |  |  |  |  |  |
| The causes and circumstances of a critical incident or error in a treatment procedure are analyzed not only at a service-level, but also at hospital-level. |  |  |  |  |  |  |
| Clinical risks are evaluated throughout the hospital. |  |  |  |  |  |  |
| Based on the cause analysis of a critical incident or error in a treatment procedure, appropriate measures are defined at hospital-level. |  |  |  |  |  |  |
| Changes in clinical risks are monitored at hospital-level. |  |  |  |  |  |  |
| The hospital-wide CRM procedures are communicated to the entire staff. |  |  |  |  |  |  |
| Reports on CRM are established for the entire hospital. |  |  |  |  |  |  |
| External specialists are involved in the further development of the hospital’s CRM (e.g. in an advisory capacity, audits, etc.). |  |  |  |  |  |  |
| **Q16 Items on leadership, participation of staff and training:** |  |  |  |  |  |  |
| CRM and questions on patient safety are regular items on the agenda of hospital board meetings. |  |  |  |  |  |  |
| The specific measures implemented by the hospital board clearly demonstrate their commitment to patient safety. |  |  |  |  |  |  |
| Staff are actively involved in CRM (e.g. by identifying clinical risks). |  |  |  |  |  |  |
| Inter-disciplinary case conferences are held at hospital-level. |  |  |  |  |  |  |
| Case conferences between professional groups are held at hospital-level. |  |  |  |  |  |  |
| Continuing training in CRM and patient safety is provided for the hospital staff on a regular basis. |  |  |  |  |  |  |

Q17 In your opinion what are the strengths of your hospital’s CRM? (open text field) ____________________________________________________________________

Q18 In your opinion where are the greatest needs for action in the CRM of your hospital? (open text field) _____________________________________________________

**2D Focal theme: Incident reporting system**

Q19 Is there a hospital-wide critical incident reporting system in your hospital (as opposed to the mandatory vigilance systems)?

 Yes, implemented in the entire hospital since (point in time when each staff group was able to or supposed to start using the system (please specify year): __________________ (go straight to Q20)

 Yes, implemented in certain services since (please specify year when implemented): __________

In how many services or areas where there is patient contact (please specify number): __________ (go straight to Q20)

 No, but is being implemented in the entire hospital (go straight to Q20)

 No, but is being implemented in certain services (go straight to Q20)

 No, but is planned for the entire hospital over the next 12 months (go straight to section 3)

 No, but is planned for certain services or areas where there is patient contact over the next 12 months (go straight to section 3)

 No (go straight to section 3)

| Q20 If there is a hospital-wide reporting system in your hospital or if you are currently in the process of implementing one for the entire hospital, please indicate to what extent the following statements apply. | Not yet examined | Examined, but so far no implementation plan | Implementation planned over the next 12 months | Not systematically implemented | Systematically implemented | Intentionally not implemented |
| --- | --- | --- | --- | --- | --- | --- |
| A definition of critical incidents that should be reported is available in the hospital or in certain services. |  |  |  |  |  |  |
| Medical malpractice claims are included in the reporting system. |  |  |  |  |  |  |
| The reporting system is computerized. |  |  |  |  |  |  |
| The reporting system is anonymous. |  |  |  |  |  |  |
| Introductory courses/training on reporting systems take place. |  |  |  |  |  |  |
| Staff get rapid feedback on critical incidents they have reported. |  |  |  |  |  |  |
| Staff are informed of reported incidents. |  |  |  |  |  |  |
| The causes of a critical incident are analyzed according to a standardized procedure. |  |  |  |  |  |  |
| Staff are informed of the results of the cause analysis. |  |  |  |  |  |  |
| Appropriate measures are defined based upon these results. |  |  |  |  |  |  |
| Staff are informed of the implemented measures. |  |  |  |  |  |  |
| The application of these measures is monitored. |  |  |  |  |  |  |

Q21 Other special features of your hospital-wide reporting system (please specify): (open text field) _____________________________________________________

**Section 3) Overview of CRM in different services**

We assume that CRM is not implemented in the same manner in all the services/departments/care centers nor in all the areas of your hospital where there is patient contact (incl. laboratories). In this section we will assess to what extent CRM key elements are being implemented or planned in the services or areas where there is patient contact. (Note: to facilitate reading, “services” will be used to describe all such areas in the questions below.)

Q22 Our hospital has ______ services/departments/care centers and other areas where there is patient contact.

(please give at least one approx. number)

| In regard to CRM key elements and their application or future implementation on a service-level, please indicate to what extent the following statements apply. | True for all services | True for certain services | Planned for all services | Planned for some services | Not true for any service |
| --- | --- | --- | --- | --- | --- |

*Multiple answers possible.*

**3A CRM Process / Q23**

| Service-internal tasks, competences and responsibilities in CRM are clearly defined. |  |  |  |  |  |
| --- | --- | --- | --- | --- | --- |
| The specific measures implemented on a regular basis by the service board of directors clearly demonstrate their commitment to patient safety. |  |  |  |  |  |
| Clinical risks are systematically identified. |  |  |  |  |  |
| The causes of clinical risks are systematically analyzed. |  |  |  |  |  |
| Clinical risks are systematically evaluated. |  |  |  |  |  |
| Measures to improve patient safety are systematically implemented. |  |  |  |  |  |
| Changes in clinical risks are systematically monitored. |  |  |  |  |  |

**3B Communication and information / Q24**

| The service’s board of directors / the department head provide a working environment where honest and open communication is encouraged. |  |  |  |  |  |
| --- | --- | --- | --- | --- | --- |
| There are guidelines (e.g. checklists, forms, procedure descriptions) to ensure that patients are informed before treatment about possible risks. |  |  |  |  |  |
| There are guidelines to ensure that patients are openly and pro-actively informed of critical incidents or errors that occurred during their treatment. |  |  |  |  |  |
| Regular surveys on treatment procedures are conducted among patients and next-of-kin. |  |  |  |  |  |
| There is an active complaints management system. |  |  |  |  |  |

**3C Documentation / Q25**

| Medical records are managed electronically. |  |  |  |  |  |
| --- | --- | --- | --- | --- | --- |
| Medical records are proactively analyzed in respect of critical incidents in treatment procedure, and appropriate measures are put in place accordingly. |  |  |  |  |  |
| There is a systematic procedure to verify whether the medical records are complete and, where necessary, to take appropriate measures. |  |  |  |  |  |

| In regard to CRM key elements and their application or future implementation on a service-level, please indicate to what extent the following statements apply. | True for all services | True for certain services | Planned for all services | Planned for some services | Not true for any service |
| --- | --- | --- | --- | --- | --- |

**3D Learning and developing / Q26**

| The service’s board of directors provides a working environment where the need for organizational and procedural improvements can be openly addressed. |  |  |  |  |  |
| --- | --- | --- | --- | --- | --- |
| The service’s board of directors takes clinical risks into account when organizational changes are implemented. |  |  |  |  |  |
| Interdisciplinary discussions on patient safety take place on a regular basis. |  |  |  |  |  |
| Staff surveys on safety culture are done on a regular basis. |  |  |  |  |  |
| Standardized procedures on the transfer of tasks and patients are applied. |  |  |  |  |  |
| Staff receive emotional support when dealing with a critical incident or error (e.g. debriefing, psychological help). |  |  |  |  |  |
| For staff that have made an error there is an opportunity for confidential discussions with co-workers. |  |  |  |  |  |

**3E Continuing education/ training/ advanced training / Q27**

| Staff are taught how to communicate when transferring tasks and patients. |  |  |  |  |  |
| --- | --- | --- | --- | --- | --- |
| Staff are trained in the early identification of critical incidents and errors. |  |  |  |  |  |
| Staff receive advanced training in effective teamwork strategies. |  |  |  |  |  |
| Staff are taught how to better evaluate their own performance (e.g. awareness of reduced alertness and fatigue). |  |  |  |  |  |
| Staff are taught how to communicate with patients regarding treatment risks and critical incidents. |  |  |  |  |  |
| Simulations are done to learn and practice how to deal with difficult procedures (e.g. practicing on the patient simulator). |  |  |  |  |  |

**3F Focal theme: Local Incident Reporting System / Q28** (If your hospital does not have a hospital-wide reporting system (see Q19-21) or if you work with different reporting systems in different services / units of your hospital.)

| A local reporting system for critical incidents has been implemented. |  |  |  |  |  |
| --- | --- | --- | --- | --- | --- |
| Training is provided on how to use the local reporting system for critical incidents. |  |  |  |  |  |
| Standardized procedures are applied for the cause analysis of reported incidents. |  |  |  |  |  |
| There is a standardized procedure for the feedback to staff on reported incidents. |  |  |  |  |  |
| The implementation of the measures derived from the cause analysis is systematically monitored. |  |  |  |  |  |

**Additional open text fields for the first survey regarding the monitoring instrument**

A1 Are there any additional themes or questions that should be included in the monitoring instrument?

(open text field) _____________________________________________________________________

A2 Do you have further comments or suggestions regarding the monitoring instrument?

(open text field) _____________________________________________________________________
